# Predicting outcome in the ICU: comparison of Ranson criteria and Ranson + CRP levels in acute pancreatitis

**DOI:** 10.1186/cc10993

**Published:** 2012-03-20

**Authors:** V Inal, L Yamanel, B Comert

**Affiliations:** 1 GATA, Ankara, Turkey

## Introduction

The aim of this study was to investigate and compare Ranson criteria (RC) and RC + serum CRP levels as a feasible, practical and precise method in acute pancreatitis (AP) cases admitted to the ICU in respect of length of stay (LOS) predicting severity of disease.

## Methods

This study was based on determination of RC scores in AP cases in a retrospective manner. On the other hand, this study included only the patients' zero-time RC scores, not the 48-hour scores, for the sake of more practical precision. Serum CRP levels were found to have prognostic importance in AP, significantly more than 150 mg/l in necrotizing AP, at 50 mg/l in this study. Therefore, patients' were evaluated for RC and RC + CRP scores for comparison. However, RC had been etiologically modified for presence of gall bladder stones (GBS); only the cases without GBS were included in order to prevent bias of results. In addition, necrotizing cases were assumed to increase CRP levels more than predicted and were also excluded. After the exclusion of cases, 89 patients' data were collected and compared for LOS in the ICU between 2005 and 2009.

## Results

Statistical analysis of patients' data for significance and receiver operating curve (ROC) analysis to predict LOS, therefore pointing to disease severity, was executed. All of the statistical comparisons were found significant for predicting LOS; RC (*P *< 0.05), RC + CRP together (*P *< 0.01) and CRP alone (*P *< 0.04). Severity of the disease and therefore LOS were increased for RC score >3 and CRP levels >50 mg/l. ROC analysis resulted in RC (AUC 0.895), RC + CRP (AUC 0.901) and CRP (AUC 0.823) for LOS.

## Conclusion

AP cases usually require ICU care and treatment. There are some consented scoring systems such as RC, APACHE II and Glasgow in predicting disease severity and guiding the physician's approach. Although the most sensitive and specific method seemed to be APACHE II scoring, it is time consuming and complex. On the other hand, RC and Glasgow scorings need to be evaluated in 48 hours. In the end, in the hardworking hours on the ICU, we need a more practical method of provision. In this study, we have found no priority of RC, RC + CRP and CRP alone in predicting AP outcome, excluding GBS disease and necrotizing cases. We conclude that, practically, ICU physicians could substantially depend on CRP levels alone in the evaluation and approach in these specific cases of AP.

